# Applications of artificial intelligence in healthcare simulation: a model of thinking

**DOI:** 10.1186/s41077-025-00379-7

**Published:** 2025-09-18

**Authors:** Adam Cheng, Carolyn McGregor

**Affiliations:** 1 KidSIM Simulation Program, Alberta Children’s Hospital, Calgary, Canada; 2 Departments of Pediatrics and Emergency Medicine, Cumming School of Medicine, University of Calgary, 28 Oki Drive NW, Calgary, AB T3B 6A8 Canada; 3 Faculty of Business and Information Technology, Ontario Tech University, Oshawa, Canada; 4 Faculty of Engineering and Information Technology, University of Technology in Sydney, Sydney, Australia

Artificial intelligence (AI) has rapidly permeated the healthcare landscape, offering a powerful tool for healthcare providers and administrators in their quest to improve the quality of care for patients [1, 2]. Healthcare simulation has similarly embraced AI, but the uptake and efforts have been largely opportunistic, with isolated demonstrations of feasibility across different areas of simulation-based education [3–5]. Simulation offers a rich, controlled environment to test and deploy AI tools while minimizing risk to patients and providers; while AI offers a powerful tool capable of transforming the way we design, deliver and evaluate simulation. In this special collection of articles in *Advances in Simulation*, we are seeking to highlight cutting-edge research, innovative methodologies, and practical applications of AI within simulation for health and social care.

AI is a broad term describing computing systems that replicate tasks or emulate mechanisms considered to require human intelligence [2]. Machine learning (ML) and deep learning refer to a subset of AI enabling the computing system to learn from data without explicitly being coded with rules and algorithms [6]. Generative AI refers to algorithms and models that can be prompted to generate many different forms of content. Within generative AI, large language models (LLMs) focus on generating text-based content. Generative AI has become more available and accessible through broadscale dissemination of LLMs through apps on handheld and other computing devices, supporting accessibility and scalability for everyday use. To fully harness the potential of AI across all areas of healthcare simulation, we must adopt a structured approach to the implementation, evaluation and adoption of AI.

## A model of thinking for AI in healthcare simulation

**Fig. 1:**
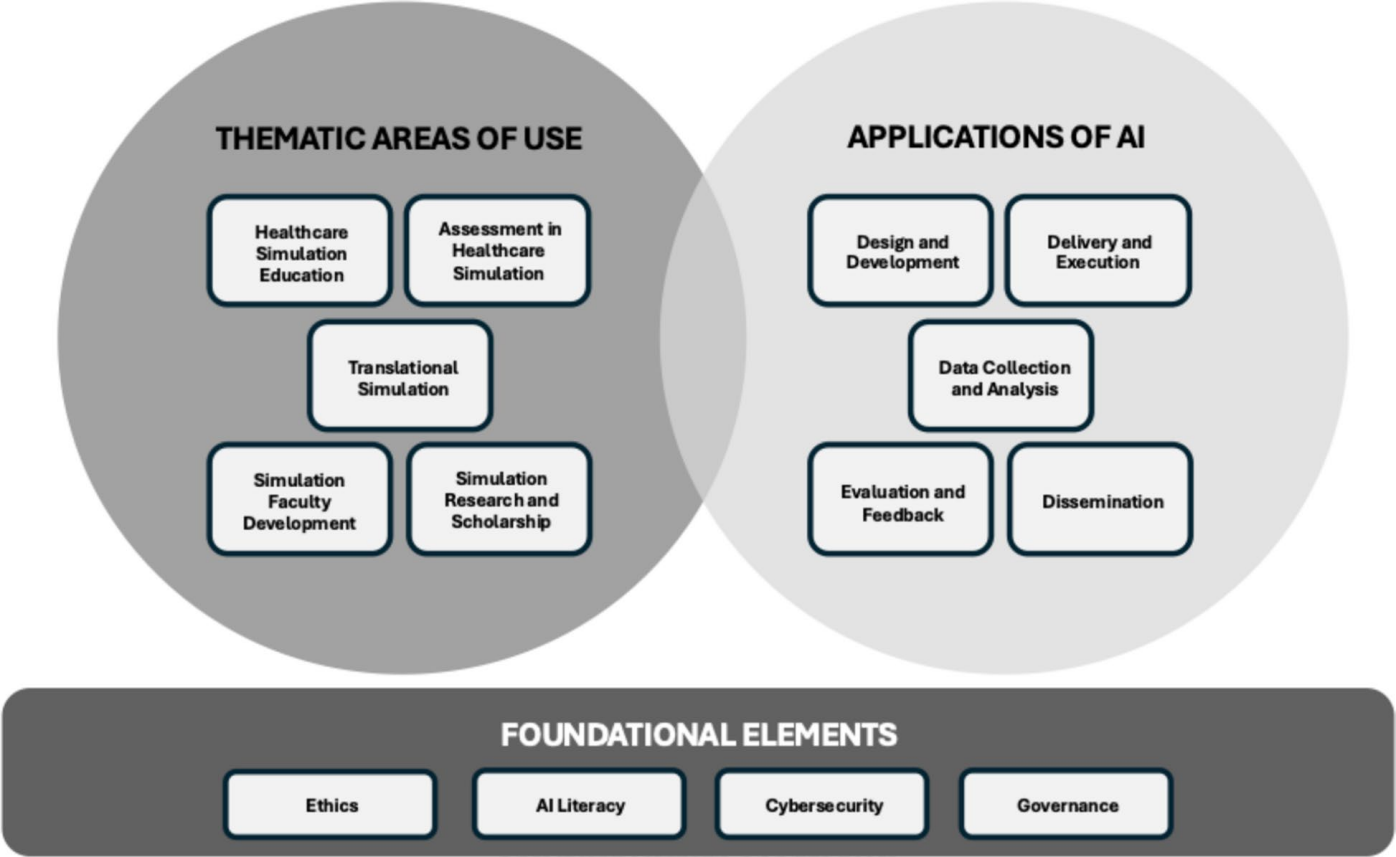
A model of thinking: applications of AI in healthcare simulation

The rapid evolution and uptake of artificial intelligence in healthcare simulation has led to its use in a variety of different ways [3–5]. As the field evolves further, it will be important to conceptualize the application of AI in an organized manner. Based on our collective experience and understanding of the published evidence, we offer the following model of thinking that illustrates how AI can be applied within healthcare simulation to advance clinical care (Fig. 1). We see five possible core thematic categories of use, many of which are closely aligned with existing evidence and established uses of simulation [7–13]. The five thematic areas of use are: (1) *Healthcare Simulation Education* [7] (i.e. training of healthcare providers and other learner groups); (2) *Assessment in Healthcare* [14] (i.e. assessing the performance of healthcare providers and other learners); (3) *Simulation Faculty Development* [15] (i.e. training simulation instructors and facilitators); (4) *Translational Simulation* [11] (i.e. use of simulation to improve patient care, patient safety, and systems-level performance); and (5) *Simulation Research and Scholarship* [12, 13] (i.e. the science of simulation; scholarship to explore why and how simulation works). While there is surely some overlap between categories, conceptualizing the application of AI across these thematic areas can help researchers and scholars think about how their work fits within the ongoing discourse of AI in healthcare simulation.

Within the five thematic areas of use, there are a handful of ways AI can be applied to support humans in achieving their project goals across these areas. First, AI can be utilized as a tool to assist with the *design and development* of simulation-based educational, research, and translational programs. For example, AI tools can be used to write learning objectives, develop simulation scenarios that are based on evidence-based clinical practice, generate debriefing outlines, adapt scenarios to learner type and brainstorm research study designs and protocols [3–5, 16–19]. Second, AI may assist with the *delivery and execution* of simulation-based interventions by being embedded within software, such as virtual patients, virtual instructors, or adaptive learning systems driven by AI [5, 20]. Third, AI can be used to *collect and analyze data* from simulations, which may allow for further insights into individual, team, or systems-level performance [4, 19, 20]. Collection of data via AI can support the fourth application, which is *evaluation of the simulation and the provision of feedback* (to learners or facilitators), which has been explored in the form of personalized feedback based upon real-time data analysis for individualised assessment and development of skill and resilience, assessments of team performance, and use of AI chatbots to guide the learning process [4, 5, 20]. Lastly, we see a role for AI in helping with the *dissemination* of simulation programs, scholarship, and research findings, such as assisting with the development of infographics and visual abstracts or providing assistance with writing [4, 20, 21].

Four foundational elements and central to the successful integration of AI in healthcare simulation programs: the ethics of AI, AI literacy, cybersecurity, and governance. A number of potential *ethical issues* should be carefully considered and addressed proactively when using AI as a tool. The inherent bias within AI systems may lead to differential outcomes for learners based on gender, race, or ethnicity; AI hallucinations (i.e. fabrication of content) may present false information affecting learning outcomes; and failure to obtain informed consent may lead to violation of data protection regulations [20–22]. Secondly, simulation programs must ensure that key stakeholders possess adequate *AI Literacy*, including an understanding of AI capabilities and limitations (e.g. ethical issues), an appreciation for how data are collected and utilized, and experience with relevant AI-driven tools developed specifically to support simulation activities [23, 24]. Successful integration of AI into healthcare simulation programs requires multifaceted techniques to protect against a broad range of *cybersecurity* threats across the spectrum of simulation applications. Strategies should be in place to address threats in areas such as (but not limited to) malware, ransomware, adversarial model manipulation, and theft of credentials or personal information [25]. Given the many areas and facets impacting the integration of AI in simulation, governance over the use of AI is paramount. Programs should consider the development of local policies for AI use, together with standardized processes for oversight, accountability, impact assessments, and ethical decision making [26].

The model of thinking for AI in Healthcare Simulation provides scholars with structure to conceptualize how they can approach the integration of AI within their simulation programs. We encourage authors to consider this model when shaping their submissions, whether they are research studies, innovations, reviews, or commentaries. In doing so, this special collection of articles in Advances in Simulation will call attention to a timely and critical opportunity: to harness AI not just as a tool to improve the efficiency of simulation, but as a catalyst for reimagining what simulation can achieve.

## Data Availability

Not applicable.
